# Drug Solubility: Importance and Enhancement Techniques

**DOI:** 10.5402/2012/195727

**Published:** 2012-07-05

**Authors:** Ketan T. Savjani, Anuradha K. Gajjar, Jignasa K. Savjani

**Affiliations:** Institute of Pharmacy, Nirma University, Sarkhej Gandhinagar Highway, Gujarat, Ahmedabad 382481, India

## Abstract

Solubility, the phenomenon of dissolution of solute in solvent to give a homogenous system, is one of the important parameters to achieve desired concentration of drug in systemic circulation for desired (anticipated) pharmacological response. Low aqueous solubility is the major problem encountered with formulation development of new chemical entities as well as for the generic development. More than 40% NCEs (new chemical entities) developed in pharmaceutical industry are practically insoluble in water. Solubility is a major challenge for formulation scientist. Any drug to be absorbed must be present in the form of solution at the site of absorption. Various techniques are used for the enhancement of the solubility of poorly soluble drugs which include physical and chemical modifications of drug and other methods like particle size reduction, crystal engineering, salt formation, solid dispersion, use of surfactant, complexation, and so forth. Selection of solubility improving method depends on drug property, site of absorption, and required dosage form characteristics.

## 1. Introduction

Solubility is the property of a solid, liquid, or gaseous chemical substance called *solute* to dissolve in a solid, liquid, or gaseous solvent to form a homogeneous solution of the solute in the solvent. The solubility of a substance fundamentally depends on the solvent used as well as on temperature and pressure. The extent of solubility of a substance in a specific solvent is measured as the saturation concentration where adding more solute does not increase its concentration in the solution [1].

The solvent is generally a liquid, which can be a pure substance or a mixture of two liquids. One may also speak of solid solution, but rarely of solution in a gas.

The extent of solubility ranges widely, from infinitely soluble (fully miscible) such as ethanol in water, to poorly soluble, such as silver chloride in water. The term *insoluble* is often applied to poorly or very poorly soluble compounds [2].

Solubility occurs under dynamic equilibrium, which means that solubility results from the simultaneous and opposing processes of dissolution and phase joining (e.g., precipitation of solids). Solubility equilibrium occurs when the two processes proceed at a constant rate. Under certain conditions equilibrium solubility may be exceeded to give a so-called supersaturated solution, which is metastable [3].

Solubility is not to be confused with the ability to dissolve or liquefy a substance, since these processes may occur not only because of dissolution but also because of a chemical reaction. For example, zinc is insoluble in hydrochloric acid, but does dissolve in it by chemically reacting into zinc chloride and hydrogen, where zinc chloride is soluble in hydrochloric acid. Solubility does not also depend on particle size or other  *kinetic *  factors; given enough time, even large particles will eventually dissolve [4].

IUPAC defines solubility as the analytical composition of a saturated solution expressed as a proportion of a designated solute in a designated solvent. Solubility may be stated in units of concentration, molality, mole fraction, mole ratio, and other units [5].

Extensive use of solubility from different perspective has led to solubility being expressed in various manners. It is commonly expressed as a concentration, either by mass (g of solute per kg of solvent, g per dL (100 mL) of solvent), molarity, molality, mole fraction, or other similar descriptions of concentration. The maximum equilibrium amount of solute that can dissolve per amount of solvent is the solubility of that solute in that solvent under the specified conditions [6]. The advantage of expressing solubility in this manner is its simplicity, while the disadvantage is that it can strongly depend on the presence of other species in the solvent (e.g., the common ion effect).

Saturated solutions of ionic compounds of relatively low solubility are sometimes described by solubility constants. It is a case of equilibrium process. It describes the balance between dissolved ions from the salt and undissolved salt. Similar to other equilibrium constants, temperature would affect the numerical value of solubility constant. The value of this constant is generally independent of the presence of other species in the solvent.

The Flory-Huggins solution theory is a theoretical model describing the solubility of polymers. The Hansen Solubility Parameters and the Hildebrand solubility parameters are empirical methods for the prediction of solubility. It is also possible to predict solubility from other physical constants such as the enthalpy of fusion.

The partition coefficient (Log P) is a measure of differential solubility of a compound in a hydrophobic solvent (octanol) and a hydrophilic solvent (water). The logarithm of these two values enables compounds to be ranked in terms of hydrophilicity (or hydrophobicity).

USP and BP classify the solubility regardless of the solvent used, just only in terms of quantification and have defined the criteria as given in Table 1 [7, 8].

The Biopharmaceutics Classification System (BCS) is a guide for predicting the intestinal drug absorption provided by the U.S. Food and Drug Administration. This system restricts the prediction using the parameters solubility and intestinal permeability.

Solubility is based on the highest-dose strength of an immediate release product. A drug is considered highly soluble when the highest dose strength is soluble in 250 mL or less of aqueous media over the pH range of 1 to 7.5. The volume estimate of 250 mL is derived from typical bioequivalence study protocols that prescribe administration of a drug product to fasting human volunteers with a glass of water [9].

The intestinal permeability classification is based on a comparison to the intravenous injection. All those factors are highly important, since 85% of the most sold drugs in the USA and Europe are orally administered.

All drugs have been divided into four classes: class I—high soluble and high permeable, class II—low soluble and high permeable, class III—low soluble and high permeable and class IV—low soluble and low permeable.

## 2. Importance of Solubility

Oral ingestion is the most convenient and commonly employed route of drug delivery due to its ease of administration, high patient compliance, costeffectiveness, least sterility constraints, and flexibility in the design of dosage form. As a result, many of the generic drug companies are inclined more to produce bioequivalent oral drug products [10].

However, the major challenge with the design of oral dosage forms lies with their poor bioavailability. The oral bioavailability depends on several factors including aqueous solubility, drug permeability, dissolution rate, first-pass metabolism, presystemic metabolism, and susceptibility to efflux mechanisms. The most frequent causes of low oral bioavailability are attributed to poor solubility and low permeability.

Solubility also plays a major role for other dosage forms like parenteral formulations as well [11]. Solubility is one of the important parameters to achieve desired concentration of drug in systemic circulation for achieving required pharmacological response [12]. Poorly water soluble drugs often require high doses in order to reach therapeutic plasma concentrations after oral administration. Low aqueous solubility is the major problem encountered with formulation development of new chemical entities as well as generic development. Any drug to be absorbed must be present in the form of an aqueous solution at the site of absorption. Water is the solvent of choice for liquid pharmaceutical formulations. Most of the drugs are either weakly acidic or weakly basic having poor aqueous solubility.

More than 40% NCEs (new chemical entities) developed in pharmaceutical industry are practically insoluble in water. These poorly water soluble drugs having slow drug absorption leads to inadequate and variable bioavailability and gastrointestinal mucosal toxicity. For orally administered drugs solubility is the most important one rate limiting parameter to achieve their desired concentration in systemic circulation for pharmacological response. Problem of solubility is a major challenge for formulation scientist [13].

The improvement of drug solubility thereby its oral bio-availability remains one of the most challenging aspects of drug development process especially for oral-drug delivery system. There are numerous approaches available and reported in literature to enhance the solubility of poorly water-soluble drugs. The techniques are chosen on the basis of certain aspects such as properties of drug under consideration, nature of excipients to be selected, and nature of intended dosage form.

The poor solubility and low dissolution rate of poorly water soluble drugs in the aqueous gastrointestinal fluids often cause insufficient bioavailability. Especially for class II (low solubility and high permeability) substances according to the BCS, the bioavailability may be enhanced by increasing the solubility and dissolution rate of the drug in the gastro-intestinal fluids. As for BCS class II drugs rate limiting step is drug release from the dosage form and solubility in the gastric fluid and not the absorption, so increasing the solubility in turn increases the bioavailability for BCS class II drugs [10, 13, 14].

The negative effect of compounds with low solubility include poor absorption and bioavailability, insufficient solubility for IV dosing, development challenges leading to increasing the development cost and time, burden shifted to patient (frequent high-dose administration) [11].

## 3. Techniques for Solubility Enhancement

Solubility improvement techniques can be categorized in to physical modification, chemical modifications of the drug substance, and other techniques.

**Physical Modifications**

Particle size reduction like micronization and nanosuspension, modification of the crystal habit like polymorphs, amorphous form and cocrystallization, drug dispersion in carriers like eutectic mixtures, solid dispersions, solid solutions and cryogenic techniques.

**Chemical Modifications**

Change of ph, use of buffer, derivatization, complexation, and salt formation.

**Miscellaneous Methods**

Supercritical fluid process, use of adjuvant like surfactant, solubilizers, cosolvency, hydrotrophy, and novel excipients.

## 4. Particle Size Reduction

The solubility of drug is often intrinsically related to drug particle size; as a particle becomes smaller, the surface area to volume ratio increases. The larger surface area allows greater interaction with the solvent which causes an increase in solubility.

Conventional methods of particle size reduction, such as comminution and spray drying, rely upon mechanical stress to disaggregate the active compound. Particle size reduction is thus permitting an efficient, reproducible, and economic means of solubility enhancement. However, the mechanical forces inherent to comminution, such as milling and grinding, often impart significant amounts of physical stress upon the drug product which may induce degradation. The thermal stress which may occur during comminution and spray drying is also a concern when processing thermosensitive or unstable active compounds. Useing traditional approaches for nearly insoluble drugs may not be able to enhance the solubility up to desired level.

Micronization is another conventional technique for the particle size reduction. Micronization increases the dissolution rate of drugs through increased surface area, it does not increase equilibrium solubility. Decreasing the particle size of these drugs, which cause increase in surface area, improve their rate of dissolution. Micronization of drugs is done by milling techniques using jet mill, rotor stator colloid mills and so forth micronization is not suitable for drugs having a high dose number because it does not change the saturation solubility of the drug [15].

These processes were applied to griseofulvin, progesterone, spironolactone diosmin, and fenofibrate. For each drug, micronization improved their digestive absorption, and consequently their bioavailability and clinical efficacy. Micronized fenofibrate exhibited more than 10-fold (1.3% to 20%) increase in dissolution in at 30 minutes biorelevant media [16, 17].

## 5. Solid Dispersion

The concept of solid dispersions was originally proposed by Sekiguchi and Obi, who investigated the generation and dissolution performance of eutectic melts of a sulfonamide drug and a water-soluble carrier in the early 1960s [18]. Solid dispersions represent a useful pharmaceutical technique for increasing the dissolution, absorption, and therapeutic efficacy of drugs in dosage forms. The term solid dispersion refers to a group of solid products consisting of at least two different components, generally a hydrophilic matrix and a hydrophobic drug. The most commonly used hydrophilic carriers for solid dispersions include polyvinylpyrrolidone (Povidone, PVP), polyethylene glycols (PEGs), Plasdone-S630. Surfactants like Tween-80, docusate sodium, Myrj-52, Pluronic-F68, and sodium lauryl sulphate (SLS) also find a place in the formulation of solid dispersion. 

The solubility of celecoxib, halofantrine, and ritonavir can be improved by solid dispersion using suitable hydrophilic carriers like celecoxib with povidone (PVP) and ritonavir with gelucire. Various techniques to prepare the solid dispersion of hydrophobic drugs with an aim to improve their aqueous solubility are listed here [19–21].

### 5.1. Hot-Melt Method (Fusion Method)

The main advantages of this direct melting method is its simplicity and economy.The melting or fusion method was first proposed by Sekiguchi and Obi to prepare fast release solid dispersion dosage forms. In this method, the physical mixture of a drug and a water-soluble carrier are heated directly until the two melts. The melted mixture is then cooled and solidified rapidly in an ice bath with rigorous stirring. The final solid mass is then crushed, pulverized, and sieved, which can be compressed into tablets with the help of tableting agents. The melting point of a binary system is dependent upon its composition, that is, the selection of the carrier and the weight fraction of the drug in the system [22].

An important requisite for the formation of solid dispersion by the hot-melt method is the miscibility of the drug and the carrier in the molten form. Another important requisite is the thermostability of both the drug and the carrier.

### 5.2. Solvent Evaporation Method

Tachibana and Nakamura [23] were the first to dissolve both the drug and the carrier in a common solvent and then evaporate the solvent under vacuum to produce a solid solution. This enabled them to produce a solid solution of the highly lipophilic *β*-carotene in the highly water soluble carrier povidone. Many investigators studied solid dispersion of meloxicam, naproxen, and nimesulide using solvent evaporation technique. These findings suggest that the above-mentioned technique can be employed successfully for improvement and stability of solid dispersions of poorly water soluble drugs [15, 17].

The main advantage of the solvent evaporation method is that thermal decomposition of drugs or carriers can be prevented because of the low temperature required for the evaporation of organic solvents. However, the disadvantages associated with this method are the higher cost of preparation, the difficulty in completely removing the organic solvent (a regulatory perspective), the possible adverse effect of the supposedly negligible amount of the solvent on the chemical stability of the drug, the selection of a common volatile solvent, and the difficulty in reproducing crystal forms [24].

### 5.3. Hot-Melt Extrusion

Hot-melt extrusion is essentially the same as the fusion method except that intense mixing of the components is induced by the extruder. Just like in the traditional fusion process, miscibility of the drug and the matrix could be a problem. High-shear forces resulting in high local temperature in the extruder is a problem for heat sensitive materials. However, compared to the traditional fusion method, this technique offers the possibility of continuous production, which makes it suitable for large-scale production. Furthermore, the product is easier to handle because at the outlet of the extruder the shape can be adapted to the next processing step without grinding [20].

## 6. Nanosuspension 

Nanosuspension technology has been developed as a promising candidate for efficient delivery of hydrophobic drugs. This technology is applied to poorly soluble drugs that are insoluble in both water and oils. A pharmaceutical nanosuspension is a biphasic system consisting of nano sized drug particles stabilized by surfactants for either oral and topical use or parenteral and pulmonary administration. The particle size distribution of the solid particles in nanosuspensions is usually less than one micron with an average particle size ranging between 200 and 600 nm [25, 26].

Various methods utilized for preparation of nanosuspensions include precipitation technique, media milling, high-pressure homogenization in water, high pressure homogenization in nonaqueous media, and combination of Precipitation and high-Pressure homogenization [27, 28].

### 6.1. Precipitation Technique

In precipitation technique the drug is dissolved in a solvent, which is then added to antisolvent to precipitate the crystals. The basic advantage of precipitation technique is the use of simple and low cost equipments; but the challenge is the addition of the growing drug crystals to avoid formation of microparticles. The limitation of this precipitation technique is that the drug needs to be soluble in at least one solvent and this solvent needs to be miscible with antisolvent. Moreover, precipitation technique is not applicable to drugs, which are simultaneously poorly soluble in aqueous and nonaqueous media [29]. Nanosuspension of Danazol and Naproxen have been prepared by precipitation technique to improve their dissolution rate and oral bioavailability. The size reduction of naproxen was also associated with an apparent increase in the rate of absorption by approximately 4-fold [30, 31].

### 6.2. Media Milling

The nanosuspensions are prepared by using high-shear media mills. The milling chamber charged with milling media, water, drug, and stabilizer is rotated at a very high-shear rate under controlled temperatures for several days (at least 2–7 days). The milling medium is composed of glass, Zirconium oxide, or highly cross-linked polystyrene resin. High energy shear forces are generated as a result of the impaction of the milling media with the drug resulting into breaking of microparticulate drug to nanosized particles [28].

### 6.3. High Pressure Homogenization

High-pressure homogenization has been used to prepare nanosuspension of many poorly water soluble drugs. In this method, the suspension of a drug and surfactant is forced under pressure through a nanosized aperture valve of a high pressure homogenizer. The principle of this method is based on cavitation in the aqueous phase. The cavitations forces within the particles are sufficiently high to convert the drug microparticles into nanoparticles. The concern with this method is the need for small sample particles before loading and the fact that many cycles of homogenization are required [32].

Dissolution rate and bioavailability of poorly soluble drugs such as spironolactone, budesonide, and omeprazole have been improved by reducing their particle size by high pressure homogenization [33–35].

### 6.4. Combined Precipitation and Homogenization

The precipitated drug nanoparticles have a tendency to continue crystal growth to the size of microcrystals. They need to be processed with high-energy forces (homogenisation). They are in completely amorphous, partially amorphous or completely crystalline forms which create problems in long term stability as well as in bioavailability, so the precipitated particle suspension is subsequently homogenized which preserve the particle size obtained after the precipitation step.

## 7. Supercritical Fluid (SCF) Process

Another novel nanosizing and solubilisation technology whose application has increased in recent years is particle size reduction via supercritical fluid (SCF) processes. Supercritical fluids are fluids whose temperature and pressure are greater than its critical temperature (Tc) and critical pressure (Tp), allowing it to assume the properties of both a liquid and a gas. At near-critical temperatures, SCFs, are highly compressible allowing moderate changes in pressure to greatly alter the density and mass transport characteristics of the fluid that largely determine its solvent power. Once the drug particles are solubilised within the SCF (usually carbon dioxide), they may be recrystallised at greatly reduced particle sizes. The flexibility and precision offered by SCF processes allows micronisation of drug particles within narrow ranges of particle size, often to submicron levels. Current SCF processes have demonstrated the ability to create nanoparticulate suspensions of particles 5–2,000 nm in diameter. Several pharmaceutical companies, such as Nektar Therapeutics and Lavipharm, are specializing in particle engineering via SCF technologies for particle size reduction and solubility enhancement. Several methods of SCF processing have been developed to address individual aspects of these shortcomings, such as precipitation with compressed antisolvent process (PCA), solution enhanced dispersion by SCF (SEDS), supercritical antisolvent processes (SAS), rapid expansion of supercritical solutions (RESS), gas anti solvent recrystallization (GAS), and aerosol supercritical extraction system (ASES) [36, 37].

## 8. Cryogenic Techniques

Cryogenic techniques have been developed to enhance the dissolution rate of drugs by creating nanostructured amorphous drug particles with high degree of porosity at very low-temperature conditions. Cryogenic inventions can be defined by the type of injection device (capillary, rotary, pneumatic, and ultrasonic nozzle), location of nozzle (above or under the liquid level), and the composition of cryogenic liquid (hydrofluoroalkanes, N_2_, Ar, O_2_, and organic solvents). After cryogenic processing, dry powder can be obtained by various drying processes like spray freeze drying, atmospheric freeze drying, vacuum freeze drying, and lyophilisation [38–40].

### 8.1. Spray Freezing onto Cryogenic Fluids

Briggs and Maxvell invented the process of spray freezing onto cryogenic fluids. In this technique, the drug and the carrier (mannitol, maltose, lactose, inositol, or dextran) were dissolved in water and atomized above the surface of a boiling agitated fluorocarbon refrigerant. Sonication probe can be placed in the stirred refrigerant to enhance the dispersion of the aqueous solution [41].

### 8.2. Spray Freezing into Cryogenic Liquids (SFL)

The SFL particle engineering technology has been used to produce amorphous nanostructured aggregates of drug powder with high surface area and good wettability. It incorporates direct liquid-liquid impingement between the automatized feed solution and cryogenic liquid to provide intense atomization into microdroplets and consequently significantly faster freezing rates. The frozen particles are then lyophilized to obtain dry and free-flowing micronized powders [42].

### 8.3. Spray Freezing into Vapor over Liquid (SFV/L)

Freezing of drug solutions in cryogenic fluid vapours and subsequent removal of frozen solvent produces fine drug particles with high wettability. During SFV/L the atomized droplets typically start to freeze in the vapor phase before they contact the cryogenic liquid. As the solvent freezes, the drug becomes supersaturated in the unfrozen regions of the atomized droplet, so fine drug particles may nucleate and grow [43].

### 8.4. Ultra-Rapid Freezing (URF)

Ultra-rapid freezing is a novel cryogenic technology that creates nanostructured drug particles with greatly enhanced surface area and desired surface morphology by using solid cryogenic substances. Application of drugs solution to the solid surface of cryogenic substrate leads to instantaneous freezing and subsequent lyophilization (for removal of solvent) forms micronized drug powder with improved solubility. Ultra rapid freezing hinders the phase separation and the crystallization of the pharmaceutical ingredients leading to intimately mixed, amorphous drug-carrier solid dispersions, and solid solutions [45].

## 9. Inclusion Complex Formation-Based Techniques

Among all the solubility enhancement techniques, inclusion complex formation technique has been employed more precisely to improve the aqueous solubility, dissolution rate, and bioavailability of poorly water soluble drugs. 

**Figure 1:**
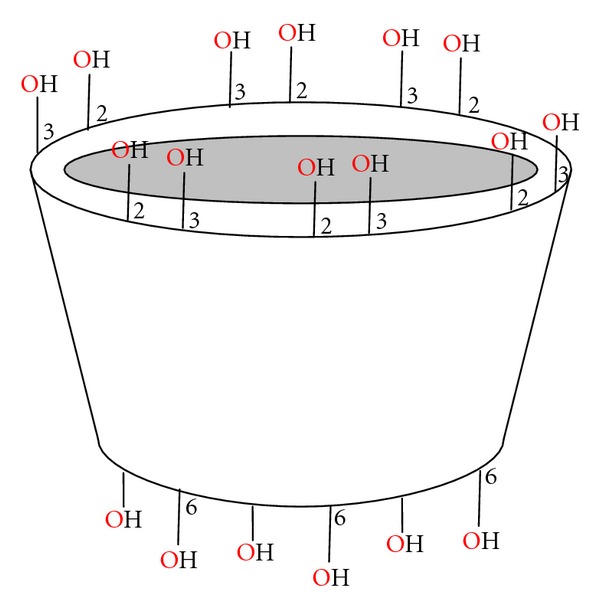
Representations of hydrophobic cavity and hydrophilic outer surface of cyclodextrin [44].

Inclusion complexes are formed by the insertion of the nonpolar molecule or the nonpolar region of one molecule (known as guest) into the cavity of another molecule or group of molecules (known as host). The most commonly used host molecules are cyclodextrins. The enzymatic degradation of starch by cyclodextrin-glycosyltransferase (CGT) produces cyclic oligomers, Cyclodextrins (CDs). These are nonreducing, crystalline, water soluble, and cyclic oligosaccharides consisting of glucose monomers arranged in a donut shaped ring having hydrophobic cavity and hydrophilic outer surface as illustrated in Figure 1. Three naturally occurring CDs are *α*-Cyclodextrin, *β*-Cyclodextrin, and *γ*-Cyclodextrin [46].

**Figure 2:**
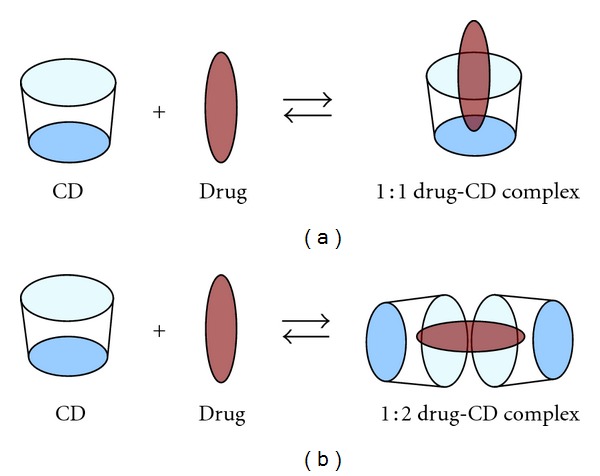
1 : 1 and 1 : 2 drug cyclodextrin complexes [47].

The surface of the cyclodextrin molecules makes them water soluble, but the hydrophobic cavity provides a microenvironment for appropriately sized non-polar molecules. Based on the structure and properties of drug molecule it can form 1 : 1 or 1 : 2 drug cyclodextrin complex as illustrated in Figure 2.

Various technologies adapted to prepare the inclusion complexes of poorly water soluble drugs with cyclodextrins are briefly described below.

### 9.1. Kneading Method

This method is based on impregnating the CDs with little amount of water or hydroalcoholic solutions to convert into a paste. The drug is then added to the above paste and kneaded for a specified time. The kneaded mixture is then dried and passed through a sieve if required. In laboratory scale, kneading can be achieved by using a mortar and pestle. In large scale, kneading can be done by utilizing the extruders and other machines. This is the most common and simple method used to prepare the inclusion complexes and it presents very low cost of production [48].

### 9.2. Lyophilization/Freeze-Drying Technique

In order to get a porous, amorphous powder with high degree of interaction between drug and CD, lyophilization/freeze drying technique is considered suitable. In this technique, the solvent system from the solution is eliminated through a primary freezing and subsequent drying of the solution containing both drug and CD at reduced pressure. Thermolabile substances can be successfully made into complex form by this method. The limitations of this technique is the use of specialized equipment, time consuming process, and yield poor flowing powdered product. Lyophilization/freeze drying technique is considered as an alternative to solvent evaporation and involve molecular mixing of drug and carrier in a common solvent [49].

### 9.3. Microwave Irradiation Method

This technique involves the microwave irradiation reaction between drug and complexing agent using a microwave oven. The drug and CD in definite molar ratio are dissolved in a mixture of water and organic solvent in a specified proportion into a round-bottom flask. The mixture is reacted for short time of about one to two minutes at 60°C in the microwave oven. After the reaction completes, adequate amount of solvent mixture is added to the above reaction mixture to remove the residual uncomplexed free drug and CD. The precipitate so obtained is separated using whatman filter paper, and dried in vacuum oven at 40°C. Microwave irradiation method is a novel method for industrial scale preparation due to its major advantage of shorter reaction times and higher yield of the product [50].

## 10. Micellar Solubilization

The use of surfactants to improve the dissolution performance of poorly soluble drug products is probably the basic, primary, and the oldest method. Surfactants reduce surface tension and improve the dissolution of lipophilic drugs in aqueous medium. They are also used to stabilise drug suspensions. When the concentration of surfactants exceeds their critical micelle concentration (CMC, which is in the range of 0.05–0.10% for most surfactants), micelle formation occurs which entrap the drugs within the micelles. This is known as micellization and generally results in enhanced solubility of poorly soluble drugs. Surfactant also improves wetting of solids and increases the rate of disintegration of solid into finer particles [11]. Commonly used nonionic surfactants include polysorbates, polyoxyethylated castor oil, polyoxyethylated glycerides, lauroyl macroglycerides, and mono- and di-fatty acid esters of low molecular weight polyethylene glycols. Surfactants are also often used to stabilize microemulsions and suspensions into which drugs are dissolved [51, 52].

Examples of poorly soluble compounds that use Micellar solubilization are antidiabetic drugs, gliclazide, glyburide, glimepiride, glipizide, repaglinide, pioglitazone, and rosiglitazone [53].

## 11. Hydrotrophy

Hydrotrophy is a solubilisation process, whereby addition of a large amount of second solute, the hydrotropic agent results in an increase in the aqueous solubility of first solute. Hydrotropic agents are ionic organic salts, consists of alkali metal salts of various organic acids. Additives or salts that increase solubility in given solvent are said to “salt in” the solute and those salts that decrease solubility “salt out” the solute. Several salts with large anions or cations that are themselves very soluble in water result in “salting in” of non electrolytes called “hydrotropic salts”; a phenomenon known as “hydrotropism.” Hydrotrophy designate the increase in solubility in water due to the presence of large amount of additives. The mechanism by which it improves solubility is more closely related to complexation involving a weak interaction between the hydrotrophic agents like sodium benzoate, sodium acetate, sodium alginate, urea, and the poorly soluble drugs [54, 55].

The hydrotropes are known to self-assemble in solution. The classification of hydrotropes on the basis of molecular structure is difficult, since a wide variety of compounds have been reported to exhibit hydrotropic behaviour. Specific examples may include ethanol, aromatic alcohols like resorcinol, pyrogallol, catechol, *α* and *β*-naphthols and salicylates, alkaloids like caffeine and nicotine, ionic surfactants like diacids, SDS (sodium dodecyl sulphate), and dodecylated oxidibenzene. The aromatic hydrotropes with anionic head groups are mostly studied compounds. They are large in number because of isomerism and their effective hydrotrope action may be due to the availability of interactive pi (*π*) orbital [56].

Hydrotropes with cationic hydrophilic group are rare, for example salts of aromatic amines, such as procaine hydrochloride. Besides enhancing the solubilization of compounds in water, they are known to exhibit influences on surfactant aggregation leading to micelle formation, phase manifestation of multicomponent systems with reference to nanodispersions and conductance percolation, clouding of surfactants and polymers, and so forth [57].

## 12. Crystal Engineering

The surface area of drug available for dissolution is dependent on its particle size and ability to be wetted by luminal fluids. This particle size, which is critical to drug dissolution rate, is dependent on the conditions of crystallization or on methods of comminution such as impact milling and fluid energy milling.

The comminution techniques can produce particles which are highly heterogeneous, charged, and cohesive, with the potential to cause problems in downstream processing and product performance. Hence, crystal engineering techniques are developed for the controlled crystallization of drugs to produce high purity powders with well-defined particle size distribution, crystal habit, crystal form (crystalline or amorphous), surface nature, and surface energy [58]. By manipulating the crystallization conditions (use of different solvents or change in the stirring or adding other components to crystallizing drug solution), it is possible to prepare crystals with different packing arrangement; such crystals are called *polymorphs*.

As a result, polymorphs for the same drug may differ in their physicochemical properties such as solubility, dissolution rate, melting point, and stability. Most drugs exhibit structural polymorphism and it is preferable to develop the most thermodynamically stable polymorph of the drug to assure reproducible bioavailability of the product over its shelf-life under a variety of real-world storage conditions. A classic example of the importance of polymorphism on bioavailability is that of chloramphenicol palmitate suspensions. It was shown that the stable polymorph of chloramphenicol palmitate produced low serum levels, whereas the metastable polymorph yielded much higher serum levels when the same dose was administered [59]. In another study, it was found that tablets prepared from the form *A *polymorph of oxytetracycline dissolved significantly more slowly than the tablets with form *B *polymorph [60]. The tablets with form *A* polymorph exhibited about 55% dissolution at 30 min, while the tablets with form *B* polymorph exhibited almost complete (95%) dissolution at the same time.

Crystal engineering approach also involves the preparation of *hydrates and solvates *for enhancing the dissolution rate. During the crystallization process, it is possible to trap molecules of the solvent within the lattice. If the solvent used is water, the resultant crystal is a hydrate; if any other solvent is used, it is referred to as solvate. The dissolution rate and solubility of a drug can differ significantly for different solvates. For example, glibenclamide has been isolated as pentanol and toluene solvates, and these solvates exhibited higher solubility and dissolution rate than two nonsolvated polymorphs [61]. It is possible for the hydrates to have either a faster or slower dissolution rate than the anhydrous form. The most usual situation is for the anhydrous form to have a faster dissolution rate than the hydrate. For example, the dissolution rate of theophylline anhydrate was faster than its hydrate form [62]. In certain cases, hydrate form of the drug may show rapid dissolution rate than its anhydrous form. Erythromycin dihydrate was found to exhibit significant differences in the dissolution rate when compared to monohydrate and anhydrate forms [63]. In general, it is undesirable to use solvates for drugs and pharmaceuticals as the presence of organic solvent residues may be toxic. Also all the organic solvents have specific limits for daily exposure to human as residual solvent in the formulated preparation.

Crystal engineering offers a number of routes to improved solubility and dissolution rate, which can be adopted through an indepth knowledge of crystallization processes and the molecular properties of active pharmaceutical ingredients. The process involves dissolving the drug in a solvent and precipitating it in a controlled manner to produce *nanoparticles *through addition of an antisolvent (usually, water) [58].

*Pharmaceutical cocrystals *open a new avenue to address the problems of poorly soluble drugs. They contain two or more distinct molecules arranged to create a new crystal form whose properties are often superior to those of each of the separate entities. The pharmaceutical cocrystals are formed between a molecular or ionic drug and a cocrystal former that is a solid under ambient conditions [64]. These are prepared by slow evaporation from a drug solution containing stoichiometric amounts of the components (cocrystal formers); however, sublimation, growth from the melt, or grinding of two or more solid cocrystal formers in a ball mill are also suitable methodologies [65].

Carbamazepine: saccharin cocrystal was shown to be superior to crystal forms of carbamazepine alone in terms of stability, dissolution, suspension stability, and oral absorption profile in dogs [66]. In another study by Childs et al., fluoxetine HCl succinic acid cocrystal was found to exhibit an approximately twofold increase in aqueous solubility after only 5 min [67]. It was observed that the itraconazole L-malic acid cocrystal exhibited a similar dissolution profile to that of the marketed formulation [68].

Traditional crystallization methods include sublimation, crystallization from solutions, evaporation, thermal treatment, desolvation, or grinding/milling. These are being replaced with novel methods of crystal engineering such as *SCF technologies* [69, 70] to produce pharmaceutical solids with desired dissolution rate and stability. *Melt sonocrystallization *is yet another emerging technology that uses ultrasonic energy to produce porous fast dissolving particles for hydrophobic drug molecules [71]. Based on these exciting reports, it appears that crystal engineering techniques need to be exploited more for enhancing the dissolution rate of poorly soluble drugs.

Other techniques that enhance the solubility of poorly water soluble drugs include salt formation, change in dielectric constant of solvent, Chemical modification of the drug, use of hydrates or solvates, use of Soluble prodrug, application of ultrasonic waves, and spherical crystallization.

## 13. Conclusion

Dissolution of drug is the rate determining step for oral absorption of the poorly water soluble drugs and solubility is the basic requirement for the absorption of the drug from GIT. The various techniques described above alone or in combination can be used to enhance the solubility of the drugs. Proper selection of solubility enhancement method is the key to ensure the goals of a good formulation like good oral bioavailability, reduce frequency of dosing and better patient compliance combined with a low cost of production. Selection of method for solubility enhancement depends upon drug characteristics like solubility, chemical nature, melting point, absorption site, physical nature, pharmacokinetic behavior and so forth, dosage form requirement like tablet or capsule formulation, strength, immediate, or modified release and so forth, and regulatory requirements like maximum daily dose of any excipients and/or drug, approved excipients, analytical accuracy and so forth.

## Figures and Tables

**Table 1:** USP and BP solubility criteria.

Descriptive term	Part of solvent required per part of solute
Very soluble	Less than 1
Freely soluble	From 1 to 10
Soluble	From 10 to 30
Sparingly soluble	From 30 to 100
Slightly soluble	From 100 to 1000
Very slightly soluble	From 1000 to 10,000
Practically insoluble	10,000 and over
